# The Significance of Arteriosclerosis in the Fundus Oculi

**Published:** 1913-02

**Authors:** 


The Significance of Arteriosclerosis in the Fundus Oculi.—Emory Hill of Chicago (*Ill. Med. Journal,* November, 1912), describes the appearance of beginning sclerosis in the retinal vessels. The early evidence of a degeneration of the arterial system, with its prophecy of a general physical and mental decay, is essential to preventive therapeutics. Modern life involves for many of the population unceasing demand on the human organism for more labor, whether it be called work or play, than it can safely perform. It is the ophthalmologist's fortune to be able to study the circulation in the retina as it can be studied nowhere else in the entire organism. The appearance of sclerotic changes here may be pathognomonic when the stethoscope, the palpating finger and the sphygmomanometer do not elicit the classis symptoms of the disease. Much has been written recently on the subject, and post mortem findings have given positive information, especially in respect to the associated vascular disease of the retina and brain, of whose nervous tissue the retina is to be regarded as an expansion. Geis, from Uhthoff's clinic in Breslau, learned that with arteriosclerosis of the retinal vessels microscopically proved there is always a similar condition of the basilar brain vessels and often localized softening of the brain tissue as well. Of 17 cases of advanced retinal arteriosclerosis all died of apoplexy, generally within four years. The vagueness of the symptoms in these cases makes them hard to treat. But their importance makes it imperative that they be not neglected. The average patient trusts more to the powers of a bitter concoction than to the pursuit of a rational hygienic plan. The problem of the overworked and overworried middle-aged man or woman is a large one; and the satisfaction is also large when we can put them in the way of health and efficiency and prolong their working years, through the combined advice of ophthalmologist and family physician, with the modicum of drugs which may serve as adjuvants to wholesome living.

